# Commentary: Atrial Fibrillation Dynamics and Ionic Block Effects in Six Heterogeneous Human 3D Virtual Atria with Distinct Repolarization Dynamics

**DOI:** 10.3389/fbioe.2017.00059

**Published:** 2017-10-06

**Authors:** Chiara Campana, Fadi G. Akar

**Affiliations:** ^1^Icahn School of Medicine at Mount Sinai, The Cardiovascular Institute, New York, NY, United States

**Keywords:** atrial fibrillation, pharmacology, rhythm control, potassium channels, repolarization

Atrial fibrillation (AF) is maintained by reentrant excitation forming stable or meandering rotors, leading circle reentry, or multiple circulating wavelets (Allessie et al., 2001; Nattel et al., 2017). While pulmonary vein triggers are critical initiators of AF (Haissaguerre et al., 1998; Pison et al., 2016), an appropriate substrate that is generated through progressive electrical and structural remodeling is required for its long-term perpetuation. Electrical remodeling comprises effective refractory period shortening, conduction slowing, wavelength reduction, and calcium-dependent triggers (Nattel, 2003; Heijman et al., 2014; Nattel et al., 2014). On the other hand, structural remodeling is hallmarked by atrial stretch and enlargement as well as interstitial fibrosis, which disrupts cell-to-cell coupling, hinders action potential propagation, and promotes reentrant excitation. Although electrical and structural remodeling is considered to be separate entities, they are highly interactive processes that influence one another during the progression of the disease. For one, fibroblast proliferation and differentiation into myofibroblasts modulates myocyte electrical function through direct coupling and paracrine signaling. Hence, a major challenge to understanding AF mechanisms is the identification of the specific contributions of electrical versus structural remodeling to fibrillatory dynamics in the disease states that promote persistent AF. Since experimentally one cannot readily separate these two processes, a rigorous computational approach is needed to isolate their individual contributions to the formation of the substrate that facilitates the maintenance of reentrant AF circuits. Multi-scale computational modeling has increasingly gained prominence in its ability to fill critical gaps that are not addressable experimentally (Cherry and Evans, 2008; Aslanidi et al., 2011; Dossel et al., 2012; Krummen et al., 2012; Colman et al., 2013; Labarthe et al., 2014; McDowell et al., 2015; Bayer et al., 2016; Boyle et al., 2016; Grandi and Maleckar, 2016; Lombardo et al., 2016; Zahid et al., 2016; Richter et al., 2017; Roney et al., 2017).

In this issue of *Front Bioeng Biotechnol*, Sanchez et al. (2017) leveraged a previously validated (Seemann et al., 2006) virtual human whole-atria model to determine how variations in the action potential morphology and repolarization gradients affect AF dynamics. Specifically, the authors constructed six electrophysiologically distinct human whole-atria models with uniform anatomical structure and fiber orientation. Electrophysiological variability in early and late repolarization was studied by incorporating cells with different action potential durations (APD) at 20, 50, and 90% of repolarization. Using an *in silico* approach that is based on experimentally calibrated human atrial action potential models, the authors confirmed key properties of AF circuits including their higher dominant frequencies in the left compared to right atria. They further provided quantitative insights to explain the predominant organization of fibrillatory activity in the regions of the pulmonary veins and right atrial appendage (Pandit and Jalife, 2013).

As expected, prolonged APD in their model impacted the organization of fibrillation patterns. Surprisingly, however, the authors identified a role for early (not just terminal) repolarization in antiarrhythmic drug therapy. AF circuits were found to be less stable and more likely to self-terminate when APD_20_ and APD_50_ were prolonged. To probe the relationship between repolarization gradients and AF dynamics, authors examined the effects of partial I_K1_, I_NaK_, and I_Na_ block on reentry organization in their virtual human atria.

This line of inquiry has clear implications for ion channel pharmacotherapy, an area of major challenge considering the suboptimal efficacy of many ion channel drugs against AF as well as their risk of inducing ventricular pro-arrhythmia. The focus on the aforementioned targets in an atria-only model is interesting from a theoretical perspective but less so from a pragmatic one. For example, *I*_Na_ blockade using Class I drugs has been extensively tested in clinical, experimental, and *in silico* studies. While these drugs are effective in treating paroxysmal AF, their efficacy may be related to suppression of triggered activity *via* non-canonical effects on RYR2 rather than *I*_Na_ (Salvage et al., 2017). More importantly, the use of flecainide in the context of persistent AF is problematic since these arrhythmias typically arise in the context of heart failure in which flecainide increases mortality (Echt et al., 1991). On the other hand, *I*_NaK_ blockade impacts nodal cell firing *via* regulation of the so-called calcium clock (Sirenko et al., 2016). As such, this approach, which mimics digitalis treatment has merit as a rate (not rhythm) control strategy. Therefore, an atria-only model is less appealing for testing the impact of *I*_NaK_ blockade than a whole-heart virtual model that incorporates neural feedback and the conduction system, simulates the ventricular response rate to AF, and tests the potential risk of proarrhythmia by digitalis toxicity. Finally, the importance of *I*_K1_ in fibrillatory dynamics is well-established. Noujaim and colleagues (Noujaim et al., 2011) demonstrated potent effects of the antimalarial drug Chloroquine in AF suppression *via* its inhibitory effects on *I*_K1_. However, this strategy must be approached with caution since *I*_K1_ density is greater in ventricular compared to atrial myocardium. A notable concern is the potential for unmasking ventricular ectopy (Miake et al., 2002) or eliciting a drug-induced form of the Andersen–Tawil syndrome (Radwanski and Poelzing, 2011).

Nonetheless, the present work by Sanchez et al. (2017) establishes a robust computational platform that should be leveraged in future studies to reveal the efficacy of promising atrial-selective channel ligands by quantifying their ability to destabilize AF circuits. For the most promising candidates, simulations should be extended to more computationally intensive, anatomically correct whole heart models that incorporate patient-specific atrial anatomy, fibrosis, and ventricular remodeling that mimic the conditions that give rise to sustained AF (Trayanova, 2011). This secondary, lower throughput strategy would allow the identification of potential pro-arrhythmic activity, which is not possible in an atria-only model. The challenge is to ensure that *in silico* studies are always constrained by strong experimental and clinical measurements to guarantee their relevance for human AF.

## Author Contributions

FA and CC drafted, revised, and approved the manuscript.

## Conflict of Interest Statement

The authors declare that the research was conducted in the absence of any commercial or financial relationships that could be construed as a potential conflict of interest.
